# Study of antibacterial activity of copper zinc nanocomposites and disruption of bacterial cytoplasmic membrane

**DOI:** 10.1038/s41598-025-93691-1

**Published:** 2025-07-23

**Authors:** Zhongshang Guo, Huihui Chen, Ruiling Hu, Jiawei Wang, Miao Wu, Yinghua Wu, Tinghui Qiang, Huan Mou, Xingguo Du, Fei Gao, Shaobo Guo, Xinli Zhou

**Affiliations:** 1 Department of Osteoarticular Surgery Department, Hanzhong Central Hospital, Hanzhong, 723000 Shaanxi China; 2 Shaanxi Key Laboratory of Catalysis, School of Chemical & Environment Science, Shaanxi University of Technology, Hanzhong, 723000 Shaanxi China

**Keywords:** ZnFe_2_O_4_, Nano-composites, Bacteriostatic materials, Antibacterial mechanism, Healing of wound, Nanoscale materials, Biomaterials - cells, Magnetic materials, Chemical biology

## Abstract

In recent years, the widespread use of antibiotics has led to the emergence of numerous drug-resistant bacteria, posing a severe threat to both human health and the economy. As a result, it is imperative to develop efficient antibacterial agents that do not induce drug resistance. This study employed layer-by-layer assembly technology to prepare ZnFe_2_O_4_@ZnS/Cu_2_S (ZZC) nanocomposites Gram-negative *Escherichia coli* (*E. coli*), Gram-positive *Staphylococcus aureus* (*S. aureus*) and drug-resistant *Salmonella* (*T-Salmonella*) were utilized as test bacteria to investigate the antibacterial effectiveness and mechanism of ZZC. The findings demonstrated that, the MIC of the ZZC against *E. coli*, *S. aureus* and *T-Salmonella* were 50, 60 and 80 μg/mL, respectively; At a material concentration of 200 μg/mL and a reaction time of 80 min, ZZC demonstrated a bacteriostatic rate of 99.99% against the three tested bacteria. The nano-composite can disrupt cell walls and plasma membranes and effectively and resulting in bacterial rupture and demise. Furthermore, the nano-composite displayed strong biocompatibility and was also able to heal mixed bacterial-induced wound infections and essentially eliminated the bacterial burden after 9 days, and also exhibited excellent antimicrobial activity in vivo. The results also indicate significant potential for its application in medical materials and other areas of research.

## Introduction

Bacterial infection presents a major health challenge for humans. Organic antibiotics have been crucial in curbing the spread of bacterial infections in recent decades^1^. Typically, organic bacteriostats target active enzymes, functional proteins, and organelles that are essential for bacterial transcription and translation to achieve their antibacterial effects. However, the overuse of antibiotics has led to the emergence of drug resistant in bacteria through external acquisition or their own evolution, resulting in a gradual loss of efficacy of the organic bacteriostats^2^. Hence, there is an urgent need for the development of effective antibacterial agents with low toxicity and the ability to inactivate drug-resistant bacteria as a substitute for organic antibiotics.

In recent years, various methods have been adopted for the preparation of metal nano-antibacterial materials. There are environmentally friendly green synthesis methods^3^, chemically stable chemical synthesis methods^4^, and the novel microfluidic synthesis technology^5^. Regarding the antibacterial performance of the prepared nanomaterials, researchers have explored the influencing factors. They have also delved into the antibacterial mechanisms, such as multi-target action mechanisms, the interaction with bacterial biofilms^4,6^, and the impacts on bacterial proteomics and gene transcription to understand how these nanomaterials inhibit bacterial growth. By evaluating the safety and toxicity of these materials, they have found wide applications in fields like food preservation and medical and health care. For example, medical dressings containing nano-copper can accelerate wound healing while inhibiting wound infections. Copper and zinc metal nanoparticles have been extensively studied due to their potent and broad-spectrum antibacterial activity, their ability to disrupt bacterial biofilms, and their status as essential trace elements for organisms^7–9^. These nanoparticles interact with negative charges on bacterial cell walls and membranes and release metal ions that alter protein secondary structures and cause irreversible damage to the bacteria. Trace metal ions can diffuse into the bacterial cell and react with various cellular organelles and components, including mitochondria, nucleic acids, and plasmids, resulting in antibacterial effects^10^. Blanca and colleagues^11^ investigated the antibacterial properties of ZnFe_2_O_4_ and showed that when dispersed in agar plates at a concentration of 27 mg/mL, it inhibited the growth of *Staphyllococcus epidermidis* by 100% and *Pseudomonas aeruginosa* by 67%. Chaliha evaluated the antimicrobial properties of different variants of Cu:ZnS nanosystems in disc diffusion assay against Gram-positive and Gram-negative bacteria. The results showed that the synthesized Cu:ZnS variants possessed good antibacterial activity^12^. Moreover, the antibacterial activity was found to increase with higher levels of Zn^2+^ and the doping of Zn^2^⁺ can also promote the generation of reactive oxygen species (ROS)^13–15^. Compared to several other nanomaterials, ZnFe_2_O_4_ nanoparticles have demonstrated notable contributions to biomedical applications, thanks to their remarkable biocompatibility^16^. But the antibacterial activity of nano-ZnFe_2_O_4_ alone is limited. Researchers, including Awais^17^ discovered that the addition of Cu particles to ZnO NP resulted in exceptional antibacterial activity against both *E. coli* and *S. aureus*. This effect was mainly due to the combination of Cu and Zn ions. Therefore, the antibacterial properties of ZnFe_2_O_4_ nanoparticles can be enhanced by compounding Cu-based antibacterial agents.

Due to their small size and large specific surface area, nanoparticles possess many unique physical, chemical, and biological properties. However, in biomedical applications, their surfaces often need to be specifically modified or treated. For example, specific chemical groups, biomolecules, or other functional substances are introduced to endow the nanoparticles with new or enhanced properties, so as to better meet the specific needs in the biomedical field^18,19^. The organic antibacterial agent ZIF-8 is a prototypic representative of zeolite-imidazolium framework (ZIFs) metal–organic frameworks (MOFs), and its exceptional performance can be applied by using it as a coating on the carrier surface^20^. The zinc (Zn^2+^) ion at the coordination center of the skeleton has powerful antibacterial activity while also serving as the source of copper for the generation of composite materials. The present study describes the development of a novel nano-composite bacteriostatic agent consisting of ZZC. The copper source was incorporated through in-situ self-assembly of ZIF-8 on the surface of the structure, followed by calcination at 200 ℃. The Gram-negative bacterium *E. coli*, Gram-positive bacterium *S. aureus* and drug-resistant bacterium Salmonella *T-Salmonella* were utilized as test bacteria. The study investigated the synergistic antibacterial efficacy and the potential antibacterial mechanism of the nanocomposites using Bordeaux solution and zineb as controls.

Metal nanoparticles, as future nanomedicines, are confronted with both opportunities and challenges^21^. In the context of wound healing, they can boost cell proliferation and tissue repair. When it comes to infection control, they possess antibacterial and antiviral properties. Thanks to their unique physical, chemical, and biological characteristics, they are utilized in bioimaging, and some magnetic metal nanoparticles can precisely and target-specifically deliver drugs under the guidance of a magnetic field. Green-synthesized metal nanoparticles have remarkable advantages. During the preparation process, they do not need external reducing agents and capping agents and generate no harmful by-products, thus being environmentally friendly and safe. However, in actual production and application, there exist issues like unstable processes and difficult quality control, rendering them unable to meet the demands of large-scale production. Moreover, many potentially therapeutic nanomaterials have not yet been approved by the US Food and Drug Administration (FDA). Currently, for biosynthesized metal nanoparticles to achieve large-scale synthesis and gain industry recognition, a substantial amount of research is still required to verify their properties and potential.

## Materials and methods

### Materials

Ferric chloride hexahydrate (FeCl_3_⋅6H_2_O) was obtained from Tianjin Damao Chemical Reagent Factory Co., Ltd. (China). Zinc chloride and ethylene glycol, anhydrous ethanol, sodium citrate and anhydrous sodium acetate were purchased from Tianjin Tianli Chemical Reagent Co., Ltd. (China). The chemical reagent, polyvinylpyrrolidone (PVP), was provided by Tianjin Obokai Chemical Co. Limitless Chemicals, Inc. (China) together with 2-methyl-imidazole, sodium disulfide nonahydrate (Na_2_S⋅9H_2_O), zinc nitrate hexahydrate (Zn(NO)_3_⋅6H_2_O), copper nitrate trihydrate (Cu(NO_3_)_2_⋅3H_2_O), tribromoethanol, phosphate buffer (PBS) (pH 7.3), propidium iodide (PI), methanol, anhydrous ethanol, Zineb, Bordeaux liquid, pancreatic peptone, agar and yeast soaking powder (Shanghai Zhanyun Chemical Co., Inc.). The products were tested on *E. coli* (BNC-C133264), *S. aureus* (BNCC186335) and *T-Salmonella* (CCTCCB 20082358) (Shaanxi Edible Fungi Research Institute). MCF-7 (Cellverse Co., Ltd.)

### Material preparation

#### Preparation of ZnFe_2_O_4_

Sixty milliliters of FeCl_3_⋅6H_2_O (0.12 mol/L) were dissolved in 60 mL of ethylene glycol and ultrasonicated uniformly, after which 60 mL of ZnCl_2_ (0.06 mol/L) was added and ultrasonicated for 30 min, after which 0.6 g sodium citrate and 3.6 g anhydrous sodium acetate were added into the reaction system and ultrasonicated for 2 h. The solution was then transferred to a reaction vessel at 200 ℃ for 10 h, after which the precipitate containing the product was magnetically separated and washed three times with distilled water and once with anhydrous ethanol and finally dried and set aside.

#### Preparation of ZnFe_2_O_4_ @ZIF-8 (ZZ)

Six hundred milligrams of ZnFe_2_O_4_ were accurately weighed out, dissolved in 150 mL of methanol, and ultrasonicated to ensure uniform mixing. PVP (9.6 g) was then added and ultrasonicated for 30 min, after which 2.6784 g of Zn(NO)_3_⋅6H_2_O was added to the supernatant and ultrasonicated for 30 min. Then, 2.9568 g of 2-methyl-imidazole was weighed into a separate beaker and dissolved in 18 mL of methanol ultrasonicated for 30 min. The 2-methyl-imidazole solution was added dropwise to the former solution, then mechanically stirred for 6 h, and separated magnetically to obtain the product. The product was washed three times with methanol, twice with anhydrous ethanol, twice with distilled water and once with anhydrous ethanol then dried for further use.

#### Preparation of ZnFe_2_O_4_@ZIF-8@ZnS (ZZZ)

Four hundred milligrams of ZZ were dissolved in 100 mL of distilled water and ultrasonicated for 20 min, after which 10 ml of Na_2_S⋅9H_2_O (0.01 mol/L) was added and stirred mechanically for 1 h, and the product was separated magnetically. The product was washed four times with distilled water and once with absolute ethanol, then dried for further use.

#### Preparation of ZnFe_2_O_4_@ZnS/Cu_2_S (ZZC)

Four hundred milligrams of ZZ were dissolved in 150 mL of methanol and sonicated for 20 min. This was followed by the addition of 40 mL of Cu(NO_3_)_2_⋅3H_2_O (0.2 mol/L) (anhydrous ethanol was used as the solvent), and the product was obtained by refluxing the product magnetically at 60 °C for 10 h. The product was washed three times with anhydrous ethanol, three times with distilled water, and dried to obtain ZnFe_2_O_4_@ZIF-8@ZnS/Cu_2_S (ZZZC). The final product was calcined at 300 ℃ for 2 h.

### Characterization

The microscopic morphology of the nanomaterials was analyzed by transmission electron microscopy (TEM) using a FEITecnai Model G2F20 instrument (FEI, The Netherlands), setting the accelerating voltage to 200 kV and the maximum magnification to 80,000 x. The dried samples were characterized by X-ray diffraction (XRD) (D8 ADVANCE, BRUKER, Germany) using Ka rays with Cu targets, an operating voltage of 40 kV, operating current of 40 mA, and scanning 2θ angle 10°–90° in 0.017°/s steps. The valence states of the elements present on the surface of the materials were analyzed using X-ray photoelectron spectroscopy (XPS, Kratos, AXIS Supra, Japan); the composition, type, and content of the elements in the prepared samples were analyzed using an energy spectrometer (JEM-F200 (HRP)), and the characteristic functional groups were detected by Fourier-transform infrared spectroscopy (VERTEX 70, Bruker, Germany). ROS was generated in the test samples by using electron paramagnetic resonance (EPR, EPR200M, Guoyi Quantum [Hefei] Technology Co., Ltd.).

### Bacteriostatic efficacy

LB solid medium was prepared using a specific ratio and autoclaved at 121 °C for 40 min (LDZF-75L, Shanghai Shen’an Medical Equipment Factory). Under aseptic conditions, single colonies of the cultured bacteria were selected and placed in test tubes containing 5 mL of culture medium. The tubes were then incubated at 37 ℃ with constant temperature and agitation for 12 h to obtain the test strains^22^. The strains were stored in a refrigerator for further use.

### Minimum Inhibitory Concentration (MIC)

The minimum inhibitory concentration of ZZC was determined using the test tube twofold dilution method. Initially, the three test bacteria were diluted to a suspension of 1.5 × 10^8^ CFU/mL. Ten sterile test tubes were prepared and numbered sequentially. Subsequently, 5 mL of the liquid culture solution and 10 μL of the suspension solution were added to each tube. The ZZC was added to 2–10 tubes, configured to a concentration of 10, 20, 30, 40, 50, 60, 70, 80 and 90 µg/mL of solution. Test tube No. 1 was designated as the control group. Subsequently, all test tubes were incubated in a constant temperature oscillating shaker at 30 °C for 12 h. The incubation was carried out at a constant temperature of 30 °C. The turbidimetric method was employed to measure the supernatant of the tubes in order, from the lowest to the highest concentration, and the BD values were recorded in three parallel sets of experiments. Prepare solutions with concentrations of 100, 110, 120, 130, 140, 150, 160, 170, and 180 μg/mL respectively, and test the MIC values of ZZ against the three types of bacteria in the same way.

### Filter paper sheet-diffusion experiment

The filter paper diffusion method was used to characterize the bacteriostatic properties of ZZC. Solutions of ZZ and ZZC with concentrations of 50, 100, 200 and 400 μg/mL were prepared in sterile deionized water. The cultured bacterial solution was diluted to 4.5 × 10^8^ colony-forming units (CFU)/mL with sterile water, applied evenly on the solid medium, and 8 µL of different materials were added dropwise on a 6 mm diameter filter paper sheet. Zinc dicentaenide (zineb) and Bordeaux solution were used as references to assess the bacteriostatic efficiency of ZZC. The experiment was repeated three times. After incubation in a 37 °C constant-temperature incubator for 12 h, the results were observed and the values of the inhibition zones were recorded. A caliper was used to measure the Zone of Inhibition (ZOI) of each in millimeters.

### Colony counting

*E. coli*, *S. aureus* and *T-Salmonella* were used as the test bacteria, and the antibacterial efficiency of ZZC was quantitatively assessed by the colony counting method. A specific amount of ZZC was added to 1.5 × 10^8^ CFU/mL suspensions of the three test bacteria. The concentration of the material was 200 µg/mL. The mixture was separated using a magnet. Then, 10 μL of the resulting supernatant was absorbed and evenly spread on LB solid medium using an inoculation ring. The contact times of ZZC with the tested bacterial solution were 0, 5, 10, 20, 40 and 80 min, respectively, and it was incubated in a constant temperature incubator at 37 ℃ for 18 h. Colony numbers were counted and the inhibition rate was calculated by applying formula (1):

1 $$\text{A}=\frac{B-C}{B}\times 100\%$$

where, A indicates the inhibition efficiency, B is the number of colonies in the control group, and C is the number of colonies resulting from inhibition of the material at different inhibition.

### Analysis of the mechanism of bacterial inhibition

#### Propidium iodide (PI) staining

The fluorescent dye propidium iodide (PI) can cross the cell membrane of damaged cells to stain double-stranded DNA within the bacterium^23^. In a sterile environment, the test bacteria (*E. coli*, *S. aureus*, *T-Salmonella*) were diluted in sterile deionized water to 1.5 × 10^8^ CFU/mL, with the test bacterial suspension serving as the control group. Eight microliters of ZZC (10 mg/mL) and 400 μL of the test bacterial suspensions were mixed well, forming the experimental group. The materials were incubated at 25 °C with shaking for 12 h. After magnetic separation, 300 μL of the supernatant was aspirated and 15 μL of PI was added for staining, then left for 10 min in the dark. The sample was then washed three times with phosphate-buffered saline (PBS, pH 6, 0.2 mol/L) and examined under an IX73 inverted fluorescence microscope (Olympus, Japan).

#### Determination of Zeta potential

A Zeta Potential analyzer was utilized to measure the potential value of the ZZC composite material and the Zeta potential of the ZZC composites against the three test bacteria (*E. coli*, *S. aureus*, *T-Salmonella*) with different reaction times. The concentrations of bacteria after overnight activation were 1.05 × 10^9^ CFU/mL, and the Zeta potential was measured as the control group. The three test bacteria were diluted and mixed with the ZZC nanocomposites at a concentration of 200 µg/mL. The mixture was left to stand for 5 and 40 min before magnetic separation. The charge on the bacterial surface was determined in the supernatant, and the data were recorded as an average of three repetitions.

#### Evaluation of cytoplasmic leakage

The leakage of bacterial contents into the solution was analyzed. Once the cell membrane of a bacterium is disrupted, the cellular endosolutes will be released into the solution and can be detected by measuring the absorbance of the solution at 260 nm using a UV–visible spectrophotometer^24^; this was used to evaluate the bacteriostatic mechanism of ZZC. After overnight growth and activation, the three test bacteria (*E. coli*, *S. aureus* and *T-Salmonella*) were diluted to 1.5 × 10^8^ CFU/mL. Four milliliters of the bacterial solutions were placed in 5 mL centrifuge tube as the controls, while ZZC nanocomposites were added to the remaining suspensions in 5 mL centrifuge tubes to concentrations of 10 mg/mL, representing the experimental samples. The mixtures were incubated for 48 h at 25 °C at room temperature with shaking, after which they were centrifuged (15,000 rpm, 10 min) and the absorbances at 260 nm were measured using a UV–visible spectrophotometer, and the data were recorded and analyzed for leakage of bacterial cytoplasmic materials in three parallel groups. Test the leakage of cellular endosolutes in ZZ in the same way.

#### Evaluation of ion leakage

Inductively coupled plasma mass spectrometry (ICP-MS) was used to detect the leakage of K^+^, Ca^2+^and Mg^2+^ ions in the bacteria to investigate the mechanism of bacterial inhibition. In the aseptic operating table, the three test bacteria were diluted according to the concentration in 2.5.2, and 3 mL of bacterial solution was added with ZZC nanocomposite (200 μg/mL) as the experimental group, and the three test bacteria were the control group. The bacterial solution of the experimental group was shaken at a constant temperature for 12 h, followed by centrifugation (15,000 r/min, 10 min), and the precipitated colonies at the bottom were dissolved in aqua regia, subjected to ablation and acid-acid-driving treatments, and then cooled down to be tested for ion leakage using the instrument (three groups were tested in parallel).

### Detection of Reactive Oxygen Species (ROS)

DMPO (5,5-dimethyl-1-pyrroline-N-oxide) was used as a spin-trapping agent for detecting hydroxyl radicals (·OH). 10 mg of each test sample (ZnFe₂O₄, ZZ, ZZZC, ZZC) was added to separate sample tubes, followed by 1 mL of distilled water. Finally, 10 μL of DMPO trapping agent was added. The solution was mixed evenly. A capillary tube (i. d. ~ 0.55 mm) was used to draw more than 5 cm of the mixed solution, which was then sealed with sealing clay. Subsequently, the sample was irradiated under a xenon lamp with a light intensity of 40% for 5 min. The capillary tube was inserted into an EPR tube and placed in a resonant cavity for testing. DMPO, with methanol as the solvent, can also be used as a spin-trapping agent for detecting superoxide anions (O₂·⁻). 10 mg of each test sample was added to separate sample tubes, along with 1 mL of methanol and 10 μL of DMPO. The solution was mixed well and oxygen was bubbled through for 1–2 min. Then, the presence of superoxide anions was tested in the same way as described above. TEMP (2,2,6,6-tetramethylpiperidine-1-oxyl) was used as a spin-trapping agent for detecting singlet oxygen (^1^O₂). 10 mg of each test material was added to separate sample tubes, followed by 19 mg of TEMP and 1 mL of ultrapure water. The solution was mixed evenly, and the operation was the same as that for detecting ·OH. All sample measurements were carried out using EPR spectroscopy at room temperature.

### Biocompatibility

Biocompatibility was assessed using the human mammary epithelial cell line MC3T3-E1. The cells were plated in 96-well plates at a density of 1500 cells per well in medium containing 10% fetal bovine serum, and 1% penicillin and streptomycin. The cells were incubated at 37 °C, saturated humidity, 5% CO_2_ and 95% air. The culture supernatants were then removed and 20 μL of nano-ZZC composites of different concentrations were added to each well and incubated under the same conditions for 24 h. Cell viability was assessed by the addition of 20 μL of MTT solution (10 mg/mL in PBS) with incubation for 4 h. The supernatant was removed and 100 μL of a methanolic lysing solution of dimethyl sulfoxide (DMSO) was added to dissolve the MTT crystals and incubated for 12 h. Absorbances at 630 nm were measured using a spectrophotometer and the toxicity of the material to the cells was assessed^25^.

### In vivo antimicrobial treatment and wound healing in animals

All animal experiments were conducted in accordance with the guidelines and regulations of the Animal Care Committee of Hanzhong Central Hospital and the study performed animal experiments in strict accordance with the ARRIVE guideline report. SD rats were provided by Hanzhong Central Hospital. The mice were anesthetized with tribromoethanol, and the mice were anesthetized and then their back hairs were removed, and a circular wound of 2.5 cm in diameter was made on the back of the mice, and a mixture of *E. coli*, *S. aureus*, and *T-Salmonella* (30 μL, 1.0 × 10^8^ CFU/mL) was injected into the skin layer of the mice, and a total of two mice were used in the control (PBS) and experimental (ZZC) groups, and the recoveries of the mice from 0 to 9 days were recorded and analysed using Image-J software to calculate the percentage of wound recovery and wound superimposition diagrams. At the same time, mice were euthanised using tribromoethanol anaesthesia overdose after 3 or 9 days of treatment, and the centre of the wound tissue was excised, soaked in saline homogenate, fixed in 4% formaldehyde solution, embedded in paraffin wax, and stained with hematoxylin–eosin (H&E) and Masson staining to observe the recovery under the microscope. All experiments were performed in triplicate. The number of mice in each group was four, and the experimental results were statistically analysed.

## Results and discussion

### Characterization of nano-ZZC composites

#### Analysis of ZZC morphology and elemental composition

**Fig. 1:**
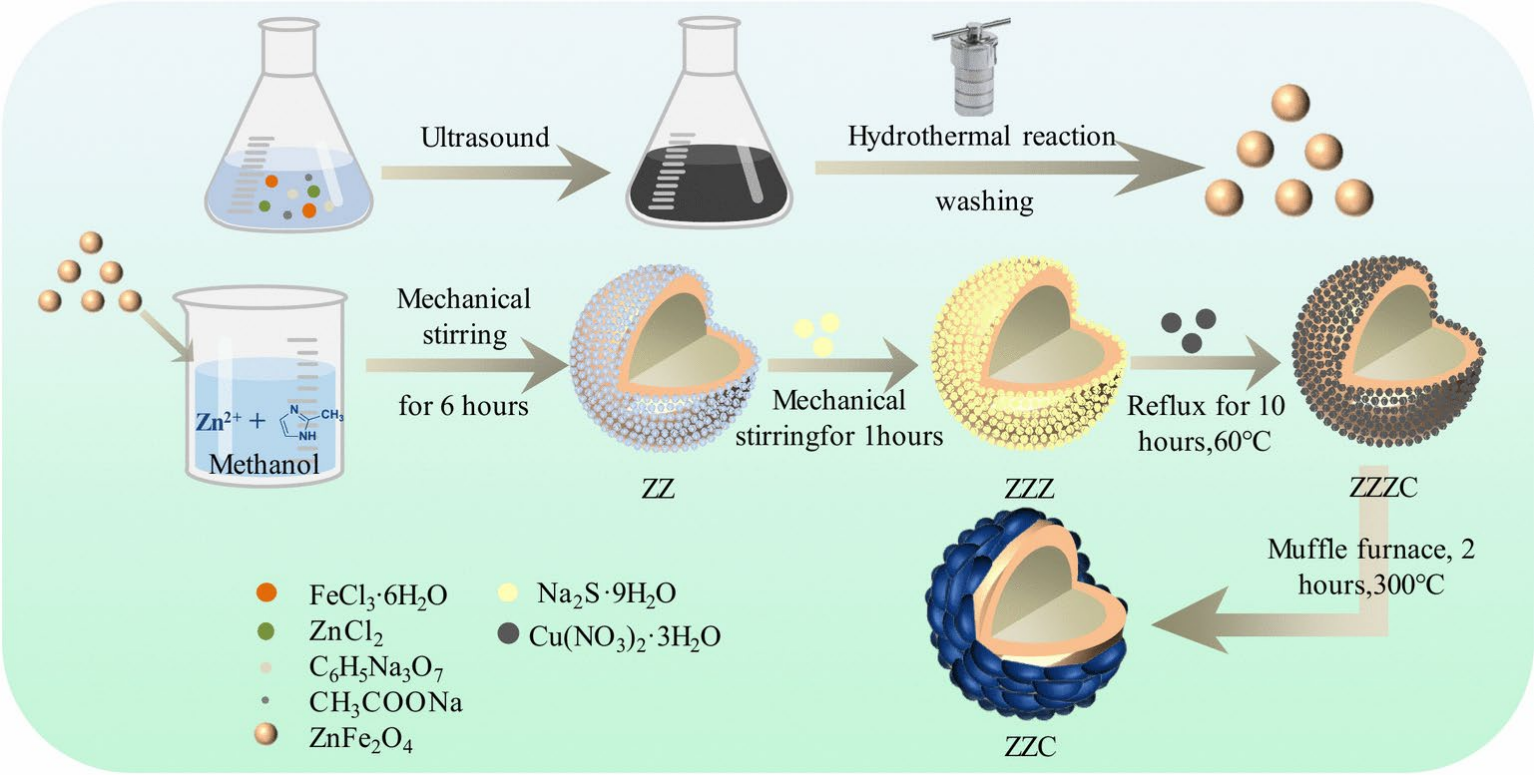
Schematic showing the synthesis of the nano- ZZC complexes.

Figure 1 shows the flow chart of the preparation of ZZC. The nanocomposite was prepared by solvothermal reaction of magnetic ZnFe_2_O_4_ particles using FeCl_3_⋅6H_2_O and ZnCl_2_ as the raw materials, sodium acetate (NaAC) and Na3Cit as stabilizers, and ethylene glycol as a reductant. After preparation of the magnetic ZnFe_2_O_4_ particles, the surface was self-assembled with ZIF-8 with the ZnFe_2_O_4_ nucleus and the Zn^2+^ coordinated to 2-methylimidazole, after which the nano-ZZC composite was obtained by obtained by the ion-exchange method and high-temperature calcination.

**Fig. 2:**
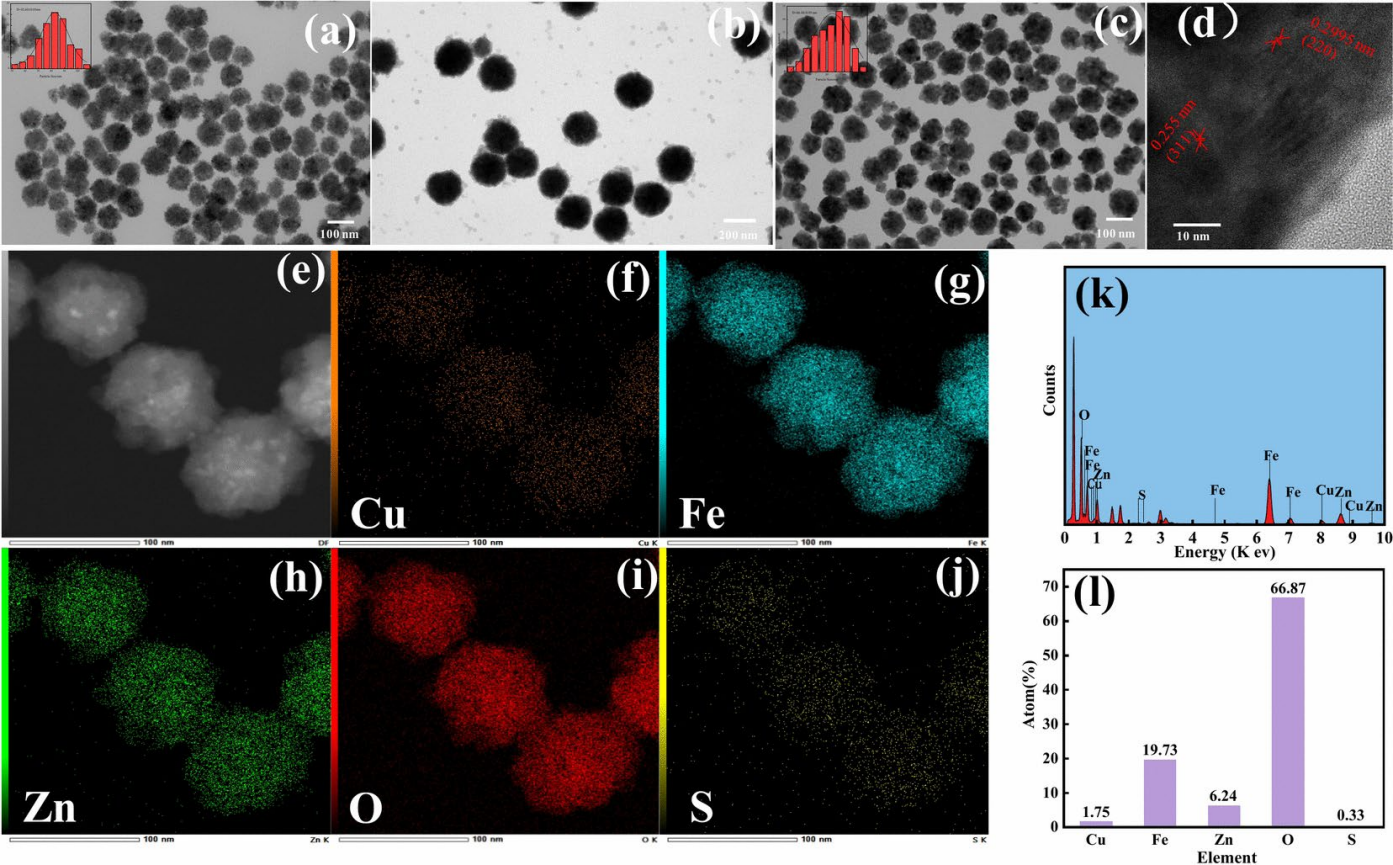
TEM image of ZnFe_2_O_4_ (**a**), ZZ (**b**) and ZZC composites (**c**). HRTEM of nano-ZZC composites (**d**). Images of EDS element mapping of nano-ZZC composites (**e**–**j**). EDS spectra and total mass fraction of the ZZC composites (**k**, **l**).

Figure 2 shows the TEM images of ZnFe_2_O_4_ and ZZC, as well as the particle size distributions. It can be seen from Fig. 2a that the monodispersed ZnFe_2_O_4_ NPs are spherical with an average particle size of 82.6 ± 0.05 nm. Figure 2b Zn-ZIF-8 thickness of 10 ± 2 nm. Figure S1 shows the uncalcined nanocomposites(ZZZC). The TEM images of the core–shell type ZZC are shown in Fig. 2c; these had an average particle size of about 86.38 ± 0.05 nm, which was slightly increased compared to the average particle size of ZnFe_2_O_4_ nanoparticles, indicating the loading of Cu_2_S on its surface. The black dots on the surface are due to the high-temperature calcination that produces the carbon elements adhering to the material surface. The HRTEM images of the ZZC are shown in Fig. 2d, and the values of the d-spacing between the layers are 0.2995 nm and 0.255 nm correspond to the (220) and (311) crystal surfaces of spinel ferrite^26,27^. To further investigate the elemental composition of the surfaces, EDS element mapping (Fig. 2e–j) and EDS energy spectra of ZZC composites (Fig. 2k) was performed, with the images of the nano-ZZC composites showing that the major elements within the composites included O, Fe, Zn, Cu and S, with atomic contents of 66.87%, 19.73%, 6.24%, 1.75% and 0.33%, respectively (Fig. 2l). In addition, the dark-field EDS elemental distribution demonstrated that O, Fe, Zn, Cu and S atoms were uniformly distributed in the shell layer.

### Analysis of ZZC crystal type and elemental valence

**Fig. 3:**
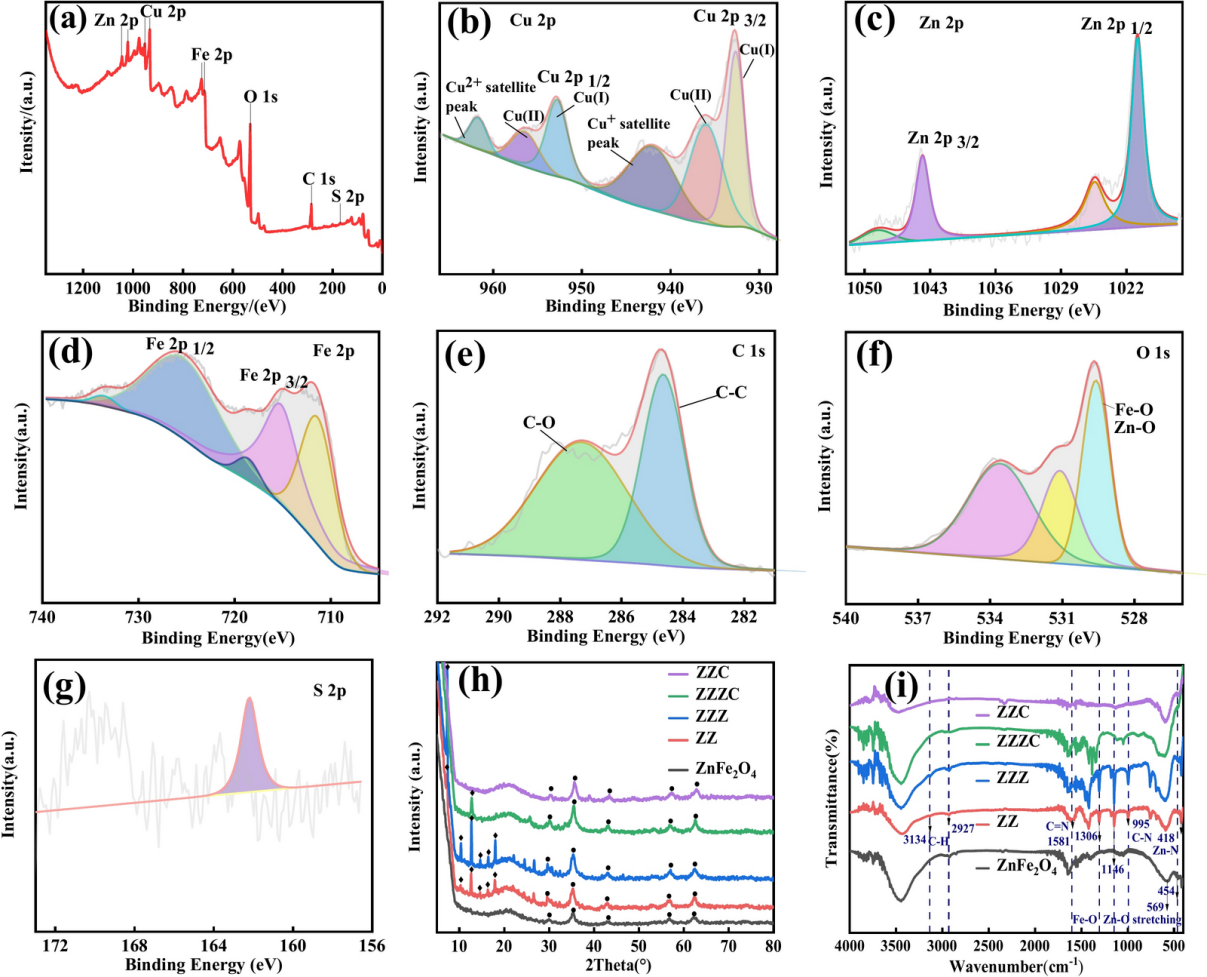
Full-scan XPS spectra of the nanocomposites (**a**). XPS spectra of Cu2p (**b**), Zn2p (**c**), Fe2p (**d**), C1s (**e**), O1s (**f**) and S2p (**g**) in nano-ZZC composites. XRD pattern of nano-ZZC composites (**h**). FT-IR spectra of nano-ZZC composites (**i**).

For further verification of the surface elements, analyses of the physical phase composition and electronic valence states of the prepared nano-ZZC composites were performed using X-ray photoelectron spectroscopy and X-ray diffraction. Figure 3a shows the full-scan XPS spectra of the nanocomposites, from which it can be seen that the characteristic peaks of the elements Cu, Zn, Fe, C, O and S are present, as shown in Fig. 3b. The two characteristic peaks located at 952.8 and 932.6 eV represent the binding energies of Cu2p_1/2_ and Cu2p_3/2_, respectively, which corresponds to the characteristic peaks of Cu^+^ in Cu_2_S, Cu^2+^ in CuS Cu2p_3/2_ and Cu2p_1/2_ binding energies^28^, and satellite characteristic peaks at 961.7 and 942.0 for Cu^2+^, which correspond to the Cu^2+^ state^29,30^. Figure 3c shows the binding energies for Zn, with 1020.8 eV and 1043.8 eV attributed to the binding energies of Zn2p_3/2_ and Zn2p_1/2_, respectively The fitted peak at 1044.9 eV is attributed to the Zn–O bond in ZnO, and the fitted peak at 1022.0 eV is attributed to the Zn2p response in ZnFe_2_O_4_ which suggests that Zn is present in the oxide in the Zn^2+^ state^31^. Figure 3d shows the characteristic peaks of Fe2p, with the binding energies at 711.4 eV and 725.2 eV attributed to Fe2p_3/2_ and Fe2p_1/2_, respectively^32^, together with two vibrational satellite peaks at 718.6 eV and 709.9 eV, demonstrating that Fe is present in ZnFe_2_O_4_ in both the Fe^3+^ and Fe^2+^ forms. In the C1s spectrum shown in Fig. 3e, elemental C is split into two peaks at 287.30 and 284.64 eV, indicating the presence of two types of carbon bonding, with the peak at 287.3 eV attributed to C-O and the peak at 284.6 eV to C–C. The O1s spectra displayed in Fig. 3f illustrate that the characteristic signals of C = O and C–O–C functional groups, are associated with the peaks at 533.5 and 531.1 eV, respectively^33^. The lattice oxygens (M–O, referred to as OLatt) in ZnFe_2_O_4_ consist of Zn–O and Fe–O bonds, as well as adsorbed oxygens or surface hydroxyl species of the catalyst (referred to as Oads)^34^. The peak at 161.2 eV in Fig. 3g is attributed to S2p_1/2_, with elemental S present in the form of S^2−^^35^.

The XRD spectra are shown in Fig. 3h. The peaks at 29.9°, 35.3°, 42.9°, 56.6°, and 62.1° correspond to the (220), (311), (400), (511) and (440) planes of ZnFe_2_O_4_, which are in general agreement with the standardized spectral position (JCPDS22-1012) of ZnFe_2_O_4_^36^. The diffraction peaks of the XRD spectra of ZZ nanocomposites showed significant diffraction peaks at 2θ = 7.3°, 10.5°, 12.8°, 14.8°, 16.5° and 18.1° compared to ZnFe_2_O_4_, which correspond to (011), (002), (112), (022), (013) and (014), respectively, especially a very long and sharp characteristic peak at 2θ = 7.3°, indicating that the high purity and crystallinity of the incorporated ZIF-8 crystals^37^. ZZ showed significantly improved crystallinity due to the increase in the Zn^2+^ content, and the lack of Cu detection in the ZZC characteristic peaks may have resulted in a Cu_2_S content below the limit of detection. The XRD spectra of ZZC after calcination showed slightly modified crystallinity due to the disappearance of some peaks caused by the carbonization of the material surface covering the internal crystalline surface.

Figure 3i shows the FT-IR spectra of ZnFe_2_O_4_, ZZ, ZZZ, ZZZC and ZZC, and it can be seen that there are obvious infrared vibrational absorption peaks of ZnFe_2_O_4_ at 454 cm^−1^ and 569 cm^−1^. These absorption peaks are characteristic of metal–oxygen bonding in the ZnFe_2_O_4_ structure^38^, with the former presenting the telescopic vibration peak of Zn^2+^-O^2−^ at the octahedral position formed by O^2−^ ions, and the latter the telescopic vibration peak of Fe^3+^-O^2−^ at the tetrahedral position formed by O^2−^ ions, indicating the presence of Zn–O and Fe–O bonding in the sample. Compared to ZnFe_2_O_4,_ the absorption peaks of ZZ at 2927 cm^−1^ and 3134 cm^−1^ are attributed to the stretching vibrations of the saturated hydrocarbon C–H(CH_3_) and unsaturated hydrocarbon C–H of 2-methylimidazolium^39^, respectively, indicating that 2-methylimidazolium plays the role of organic ligands in the nanocomposites. The peak at 1581 cm^−1^ represents C=N stretching vibrations, with C–N stretching vibrations at 1146 cm^−1^ and 995 cm^−1^; the peak at 1306 cm^−1^ is attributed to imidazolium ring vibration, and the peak at 418 cm^−1^ is caused by Zn-N bond stretching vibration. These findings demonstrate the successful encapsulation of ZIF-8 in ZnFe_2_O_4_. The FT-IR spectra of ZZZ showed no significant changes in the peak positions compared to ZZ. The characteristic peaks of C=N in ZZZC have shifted to the left, suggesting that there may be an interaction between copper and nitrogen^40^. Some characteristic peaks of ZZC disappear after calcination.

### Bacteriostatic efficacy of nano-ZZC composites

#### MIC

Figure S2 shows the experimental results of the MIC of ZZ against *E. coli*, *S. aureus*, and *T-Salmonella*. The MIC values are 120, 120, and 130 µg/mL respectively. The results of the MIC tests for different concentrations of ZZC on the three test bacteria, *E. coli*, *S. aureus* and *T-Salmonella*, are presented in Fig. S2. *E. coli* was successfully removed from test tube No. 6, at which time the value of the turbidimeter test was 0.25 (Table S1), and that of the control group No.1 was 5.73, the concentration of the bacterial solution subsequently decreased by approximately 96%. This indicated that the MIC value of the composites on *E. coli* was 50 µg/mL, and similarly for *S. aureus* and *T-Salmonella*, the MIC values were 60 and 80 µg/mL, respectively. Compared with other studies, ZZC has lower minimum inhibitory concentration (MIC) values^41^. After the introduction of copper ions, the antibacterial performance of ZZC is significantly stronger than that of ZZ. The inhibitory effect of ZZC on the three tested bacteria was observed to be greatest against *E. coli*, with a lesser effect observed against *S. aureus* and *T-Salmonella*.

#### Filter paper sheet diffusion method

**Fig. 4:**
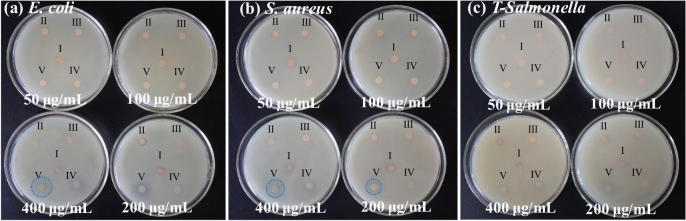
Results of filter paper diffusion of different concentrations of ZnFe_2_O_4_, ZZ, ZZZ, ZZZC and nano-ZZC composites against *E. coli* (**a**), *S. aureus* (**b**) and *T-Salmonella* (**c**).

The bacteriostatic effects of the ZZC nanocomposite on *E. coli*, *S. aureus*, and *T-Salmonella* typhimurium were determined using the filter paper disk diffusion method. In Fig. S3a–c, I and II (zineb and Bordeaux solution, respectively) were used as controls, III and IV (ZZ and ZZC, respectively) were used as the experimental groups. When the concentration was 50 μg/mL, no obvious inhibition zones were observed in all four samples. At concentrations of 100, 200 and 400 μg/mL, ZZC produced larger inhibition zones compared with zineb and Bordeaux mixture at the same concentrations. From the line graph of the inhibition zone diameters in Fig. S3d–f, it can be seen that 200 μg/mL is the optimal bacteriostatic concentration within this concentration range. Figure 4 shows the filter-paper disk diffusion results of each precursor of the nanomaterial and ZZC, where I, II, III, IV and V represent zinc ferrite, ZZ, ZZZ, ZZZC, and ZZC respectively. Table 1 shows the table of inhibition zone diameters of each precursor and ZZC at different concentrations. As can be seen from the table, when the concentration is 400 μg/mL, the inhibition zones of the ZZC composite material against *E. coli*, *S. aureus*, and *T-Salmonella* are 18, 16 and 9 mm respectively. The antibacterial efficiency of the ZZC composite material against *E. coli*, *S. aureus*, and *T-Salmonella* have increased by 2.25, 2.00 and 1.5 times respectively compared with ZZ, and by 2.12, 2.13 and 1.5 times respectively compared with ZZZ. In conclusion, the bacteriostatic performance of this composite is significantly stronger than that of commercially available inorganic bacteriostatic compounds and each precursor component. Among them, the ZZC nanocomposite exhibits stronger inhibitory performance against *E. coli* than against *S. aureus* and *T-Salmonella*, indicating that the bacteriostatic effect of this nanocomposite has been significantly enhanced after the introduction of copper ions, and it has a higher inhibitory activity against the Gram-negative bacterium *E. coli*.

**Table 1:** Zone of inhibition values (mm) of nano-ZZC composites with each precursor against *E. coli*, *S. aureus* and *T-Salmonella*.

Bacterial	c (μg/mL)	Different materials (days) (± 0.5 mm)				
		Z	ZZ	ZZZ	ZZZC	ZZC
*E. coli*	50	6	6	6	6	6
	100	6	6	6	6.5	8.5
	200	6	6.5	6.5	8.5	15
	400	6.5	8	8.5	10	18
*S. aureus*	50	6	6	6	6	6
	100	6	6	6.5	7	7.5
	200	6	6.5	7	8	14
	400	7	8	7.5	9.5	16
*T-Salmonella*	50	6	6	6	6	6
	100	6	6	6	6	6.5
	200	6	6	6	6.5	8
	400	6	6	6	7.5	9

### Colony-counting method

**Fig. 5:**
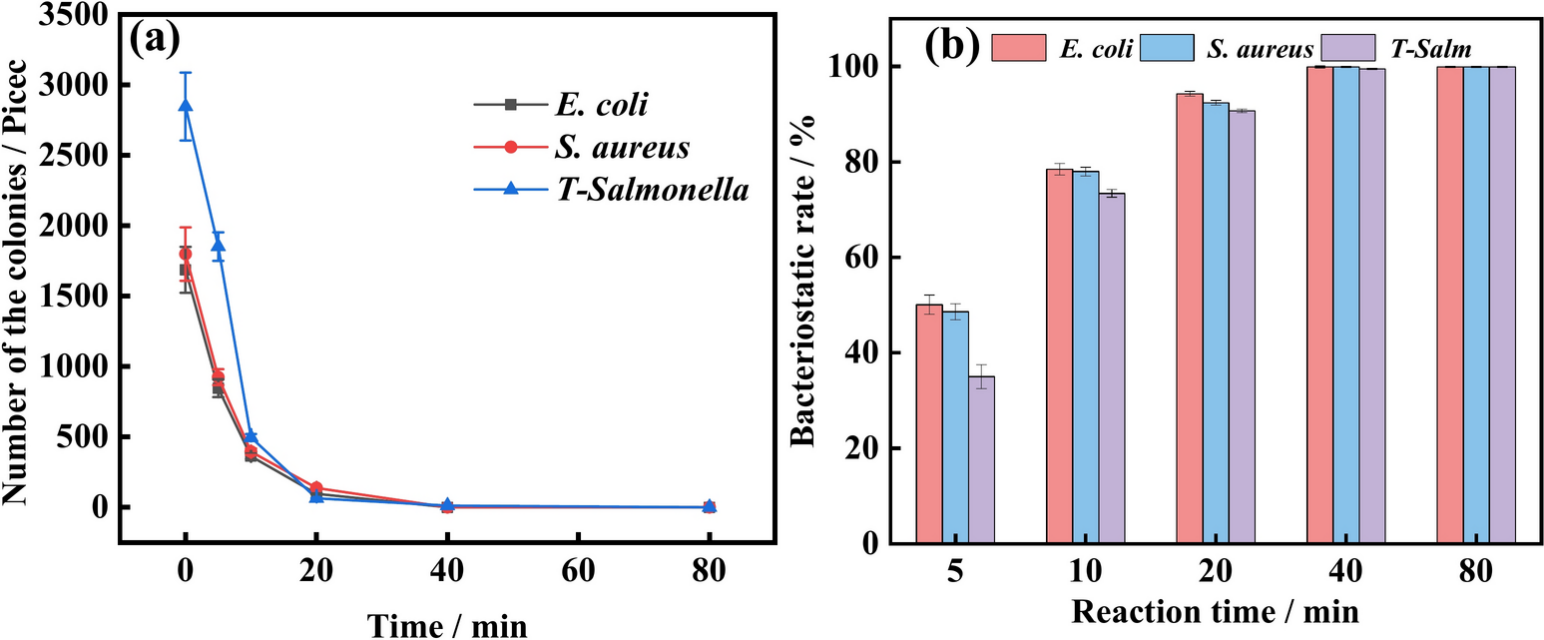
(**a**) Colony numbers at different times after treatment of the three test bacteria with nano-ZZC composites. (**b**) Bacteriostatic rates of nano-ZZC composites against the three test bacteria at different times.

To further investigate the antibacterial properties of ZZC nanocomposites, we analyzed the antibacterial efficacy of the composites at a concentration of 200 µg/mL after mixing with *S. aureus* (Fig. S5a), *E. coli* (Fig. S5b), and *T-Salmonella* (Fig. S5c). The colony-counting method was utilized to determine antibacterial activity at 0, 5, 10, 20, 40 and 80 min. 0 min indicates the control group and as can be seen from the figure, increased mixing time was associated with greater inhibition of the growth of the three test bacteria, and the inhibitory effects of the nanocomposites on the three test bacteria, *E. coli*, *S. aureus* and *T-Salmonella*, were obviously enhanced. Figure 5a shows the number of colonies observed after mixing the three test bacteria with the material for different times, and Fig. 5b shows the inhibition rate of ZZC nanocomposites against the bacteria demonstrating inhibition rates of 50.1%, 48.6% and 35%, after 5 min incubation with *E. coli*, *S. aureus* and *T-Salmonella*, increasing after 10 min to 78.5% in *E. coli*, while the inhibition of *S. aureus* and *T-Salmonella* reached 78% and 73.4%, respectively. By 80 min, the rate of inhibition of all three bacteria was over 99%.

### ***Mechanism of bacterial inhibition by nano-ZnFe***_***2***_***O***_***4***_***@ZnS/Cu***_***2***_***S composites***

#### Analysis of the results of PI staining

**Fig. 6:**
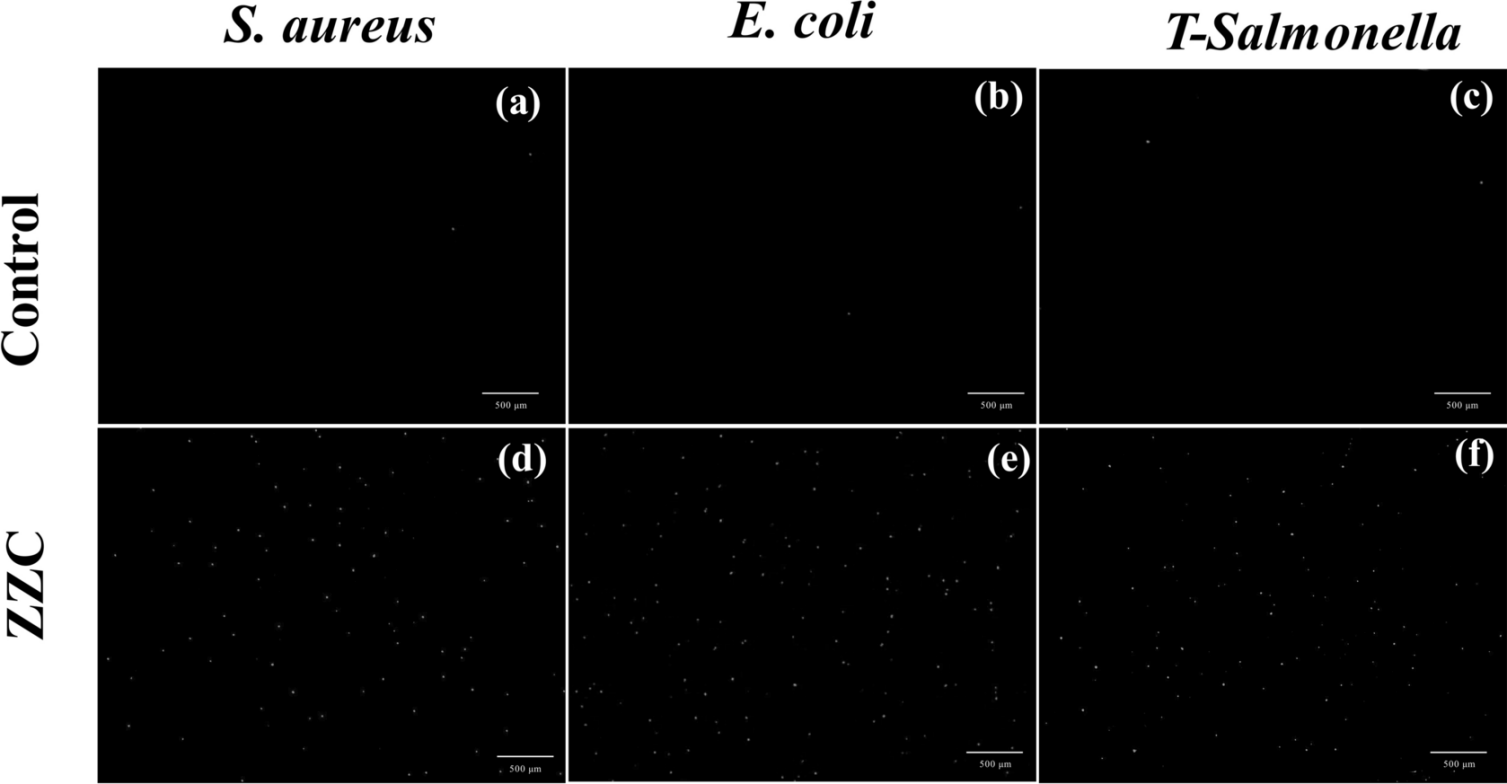
Three test bacteria not treated with nano-ZZC (**a**–**c**). Representative fluorescence images of bacterial cells after 12 h of treatment. Dead *S. aureus* (**d**), *E. coli* (**e**) and *T-Salmonella* (**f**), shown by PI staining.

The fluorescent dye PI stains the DNA of dead cells, resulting in the nuclei of dead cells emitting red fluorescence when examined under fluorescence microscopy^24,42^. As can be seen in Fig. 6a–c, a few bacteria in the blank control group of the pure bacterial suspensions of *E. coli*, *S. aureus* and *T-Salmonella* showed red fluorescent dots after PI staining, indicating low numbers of dead bacteria. However, after incubation of the ZZC composite with the test bacteria for 12 h, followed by 10 min PI staining, all three bacteria showed greater numbers of red fluorescent dots, with the most seen for *E. coli* (Fig. 6e), indicating that the composite caused the most serious membrane damage to *E. coli*, followed by *S. aureus* (Fig. 6d), while the effect was weaker for the drug-resistant bacterium *T-Salmonella* (Fig. 6f). The experimental results showed that the nano-ZZC composite could effectively damage the bacterial cell membrane.

#### Zeta potential analysis

**Fig. 7:**
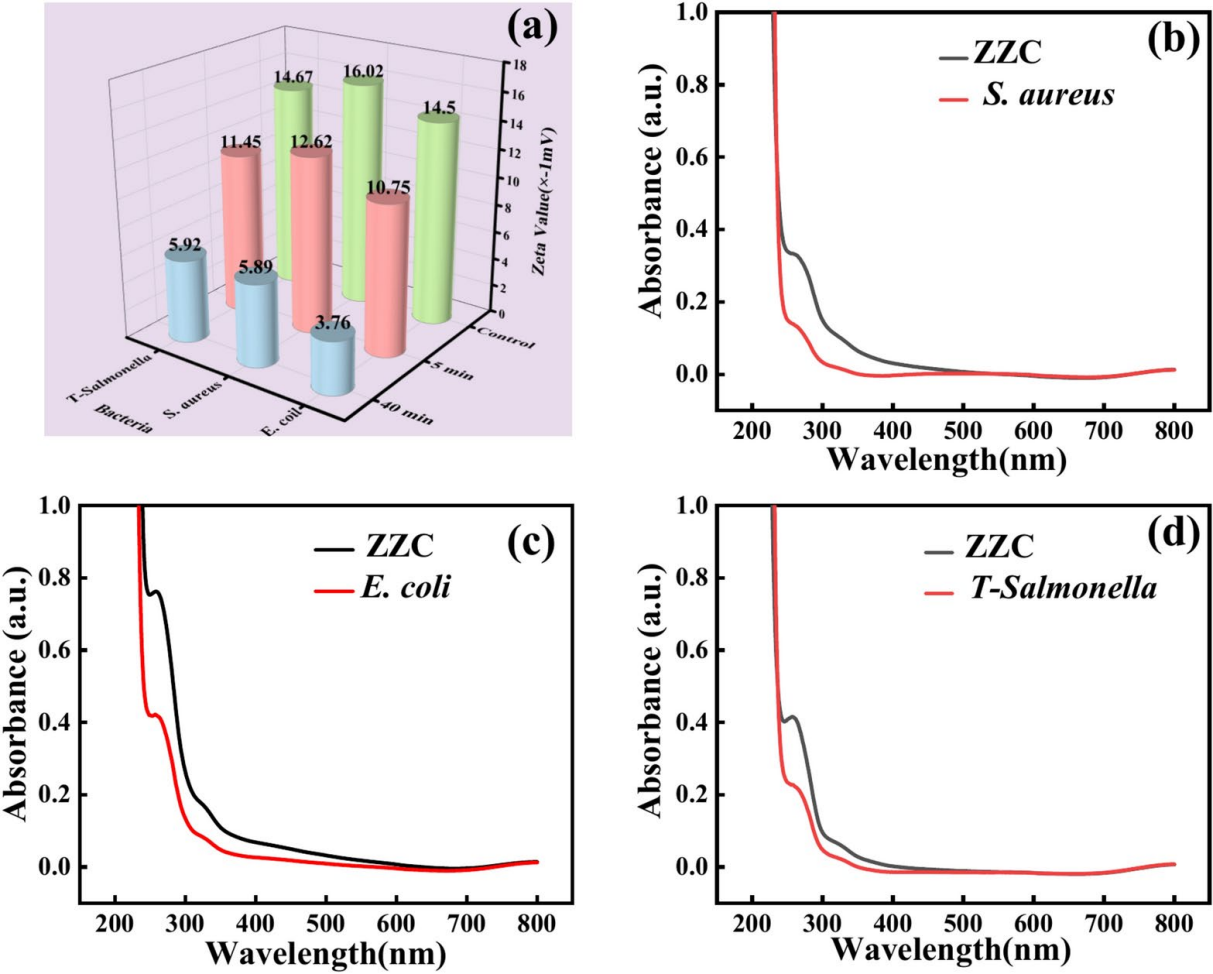
Zeta potentials of 200 µg/mL nano-ZZC composites mixed with *S. aureus*, *E. coli* and *T- Salmonella* for 5 and 40 min (**a**). Cytoplasmic leakage of *S. aureus* (**b**), *E. coli* (**c**) and *T-Salmonella* (**d**) after treatment with the nanocomposites.

Zeta potential measurements can effectively assess the degree of mutual repulsion or attraction between nano-materials within biological systems and the environment, as well as reveal the surface charge distribution of cell membranes^30^. Figure S6 shows that the surface potential of the ZZC composite material is − 36.82 mV. Figure 7a demonstrates the impact of mixing 200 µg/mL of nano-ZZC composite with *E. coli*, *S. aureus* and *T-Salmonella* for 5 and 40 min on the Zeta potential values of the bacterial cell membranes. Using the Zeta potential values of pure *E. coli*, *S. aureus* and *T-Salmonella* as the controls, the values for the surface potentials of the three tested bacteria were − 14.50, − 16.02 and − 14.67 mV, respectively. Since lipopolysaccharides and lipoproteins in the bacterial cell wall contain negatively charged reactive groups such as –OH, –COOH, –CONH_2_ and –NH_2_. The figure shows that the surface potentials of nano-ZZC composites after mixing with *E. coli*, *S. aureus* and *T-Salmonella* for 5 min were − 10.75, − 12.62 and − 11.45 mV, respectively. Subsequently, after 40 min, the surface potentials of the three bacteria were − 3.76, − 5.89 and − 5.92 mV, respectively. The nano-ZZC can effectively neutralise the surface charge of bacterial cell walls and have a more pronounced effect on *E. coli*.

#### Analysis of the results of Cytoplasmic leakage and ion leakage

Cytoplasmic leakage was used for further investigation of the bacteriostatic mechanism of the nano-ZZC composites against the bacteria. Damage to microbial cell membranes leads to the release of intracellular components, including DNA, RNA, plasmids, and other biomolecules, into the surrounding environment. The absorption peaks of the released materials were measured at 260 nm using a UV–visible spectrophotometer. The outcomes are depicted in Fig. 7. Compared to the control group, the absorbance at 260 nm of *S. aureus* (Fig. 7b), *E. coli* (Fig. 7c) and *T-Salmonella* (Fig. 7d) demonstrated a notable increase in the absorbance at 260 nm after exposure to the nano-ZZC composite material. Moreover, compared with ZZ (Fig. S7), the leakage of macromolecules was also significantly higher. This significant increase is indicative of the substantial leakage of the bacterial cellular contents.

**Fig. 8:**
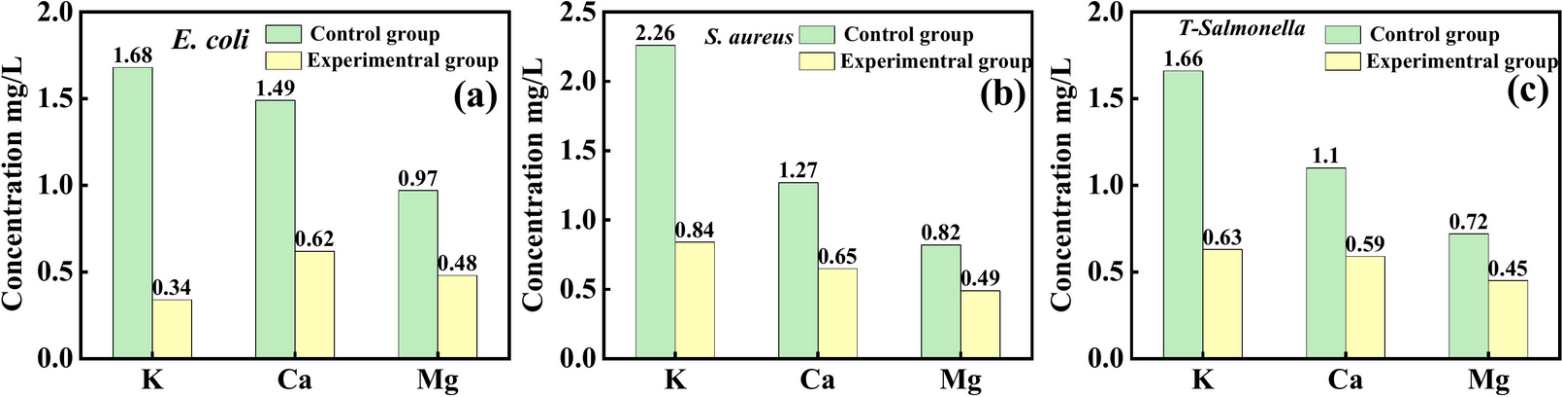
The results of Ion leakage of *E. coli* (**a**), *S. aureus* (**b**) and *T-Salmonella* (**c**).

Damage to the bacterial cell membrane leads to leakage of intracellular ions, which results in metabolic disorders 0. In this experiment, the degree of change in cell membrane permeability of three test bacteria, *E. coli*, *S. aureus* and *T-Salmonella*, by ZZC nanocomposites was further evaluated by determining the leakage of K^+^, Ca^2+^, and Mg^2+^ in the bacteria. Figure 8 shows the results of the effect of composites on the leakage of intracellular K^+^ (Fig. 8a), Ca^2+^ (Fig. 8b), and Mg^2+^ (Fig. 8c) of the three test bacteria, where the concentrations of intracellular K^+^ in *E. coli*, *S. aureus*, and *T-Salmonella* in the control group were 1.68, 2.26, and 1.66 mg/L, respectively; the concentrations of Ca^2+^ were 1.49, 1.27, and 1.10 mg/L, respectively and the concentrations of Mg^2+^ were 0.97, 0.82 and 0.72 mg/L, respectively, indicating that the bacterial cells maintained their own normal ion channels. After a period of time of ZZC action, the concentrations of intracellular K^+^ in *E. coli*, *S. aureus* and *T-Salmonella* were 0.34, 0.84 and 0.63 mg/L, respectively, and those of Ca^2+^ were 0.62, 0.65 and 0.59 mg/L, and the concentrations of Mg^2+^ were 0.48, 0.49 and 0.45 mg/L, respectively, which were significantly lower than those of the control. The bacterial cytoplasmic membrane serves as a permeability barrier for ions and most molecules and regulates the channels through which these substances enter and exit the cell^43^. Before the addition of the nanomaterials, the cells maintain their own ion channels. After adding ZZC, the experimental results show that the composite material can disrupt the ion channels on the bacterial cell membrane, causing a large amount of K⁺, Ca^2^⁺, and Mg^2^⁺ to leak out. ZZC materials can effectively destroy the normal physiological functions of bacteria, resulting in the disruption of their ion channels.

#### ROS generation and detection

**Fig. 9:**
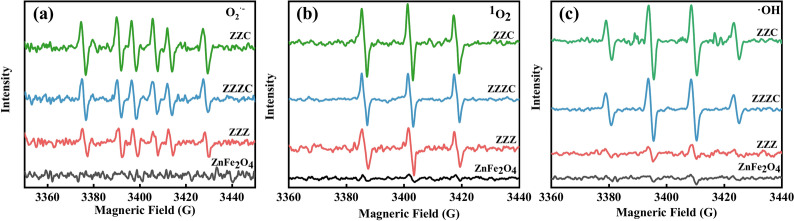
Different samples were tested using DMPO, DMPO + CH₃OH, and TEMP as spin traps to obtain the Electron Paramagnetic Resonance (EPR) spectra of O_2_^·−^, ^1^O₂, and ·OH.

The EPR technique was employed to detect the generation of ROS in different samples. Figure 9a shows the characteristic spectrum of the spin adduct formed between DMPO/MeOH and O_2_^·−^. The resulting image exhibits a sextet spectrum, indicating the presence of superoxide in the system. Figure 9b presents the EPR spectrum of TEMP. As a trapping agent for ^1^O₂, TEMP reacts with ^1^O₂ to generate a nitroxide radical with a peak intensity ratio of 1:1:1. In Fig. 9c, the EPR signal of ⋅OH trapped by DMPO can be clearly observed. It is a quartet spectrum with a ratio of 1:2:2:1, which is characteristic of the DMPO-⋅OH adduct. Electrons generated by ZnFe₂O₄ under xenon lamp irradiation can react with molecular oxygen to produce O_2_^·−^. H⁺ on the surface of ZnFe₂O₄ can extract electrons from water and hydroxyl ions in ZnFe₂O₄, generating ⋅OH through an oxidation process^44^. ^1^O₂ is mainly produced indirectly by the aqueous reaction of O₂. Additionally, bacterial cells can produce abundant H₂O₂, which can be activated by Fe^3^⁺/Fe^2^⁺ through the Fenton reaction to generate toxic ⋅OH. Therefore, these three types of ROS can be detected in ZnFe₂O₄^45^. When more zinc ions and copper ions are introduced into the nanomaterial, a large number of these ions play a role in promoting the generation of ROS and the Fenton reaction, increasing the relative intensities of the spectra of O₂·⁻, ^1^O₂, and ⋅OH. The high ROS production can induce oxidative stress in bacterial cells, improving the antibacterial performance of the nano-ZZC composites material.

#### Cytotoxicity result analysis

**Fig. 10:**
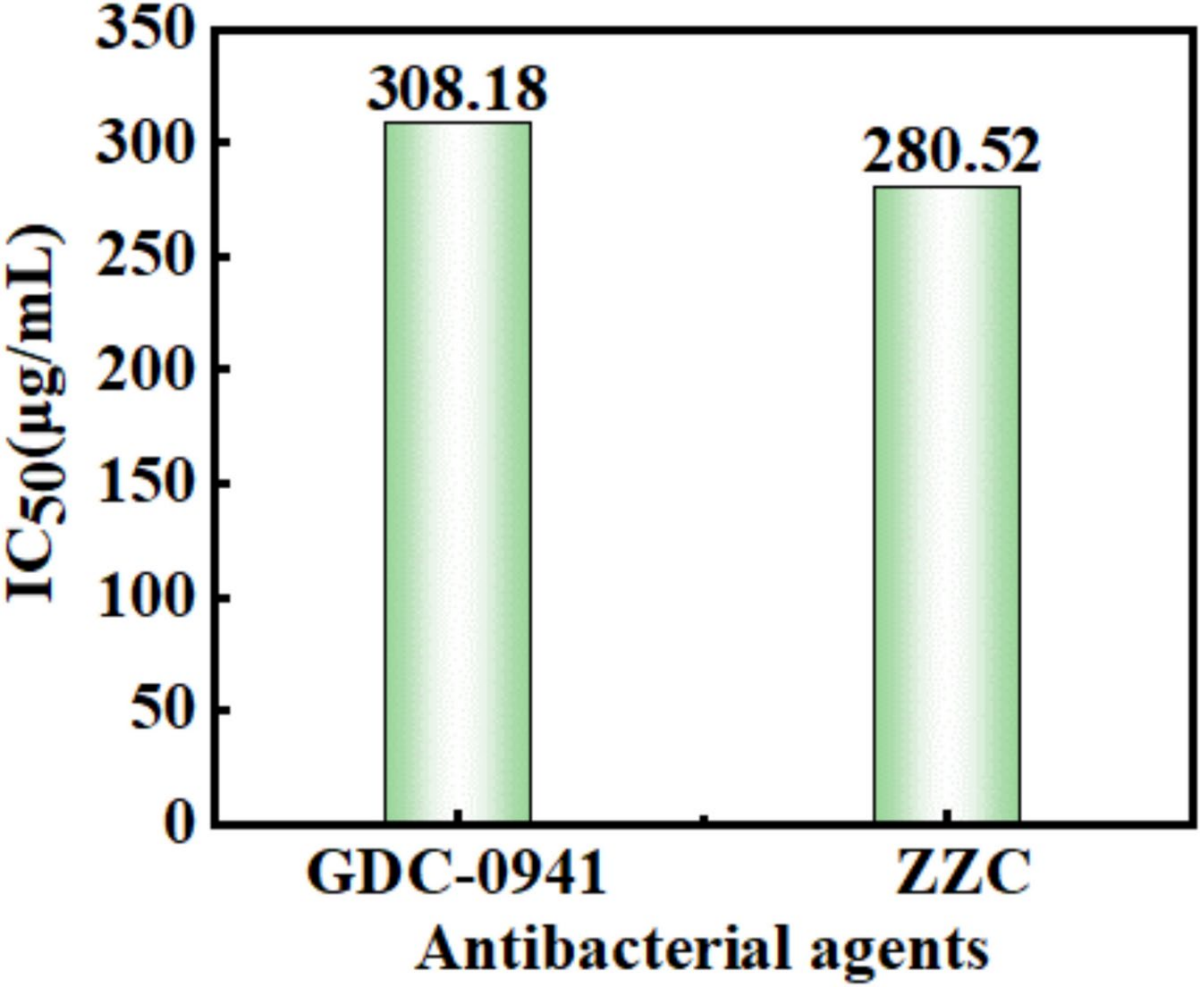
Toxicity analysis of the ZZC composites.

Biocompatibility is critical for nanomaterials to be applied in biomed-icine^7,46^. Therefore, the biocompatibility of the nano-ZZC composites was evaluated by comparing the toxicities of nano-ZZC composites and GDC-0941 anticancer composites on MC3T3-E1 mouse-derived fibroblast cells, which were cultured with different drugs for 24 h. The results are shown in Fig. 10. The IC_50_ values, compared with the negative control group, of GDC-0941 and ZZC were 308.18 and 280.52 (± 0.05) μg/mL, respectively, which showed no statistically significant difference and the IC₇₀ (Fig. S8) value of ZZC was 408.60 µg/mL (± 0.05). It can be seen that the biocompatibility of the ZZC nanocomposites is similar to that of the commercially available anticancer drug GDC-0941, demonstrating the good biocompatibility of the nanocomposites.

### In vivo antibacterial and wound healing properties of ZZC

**Fig. 11:**
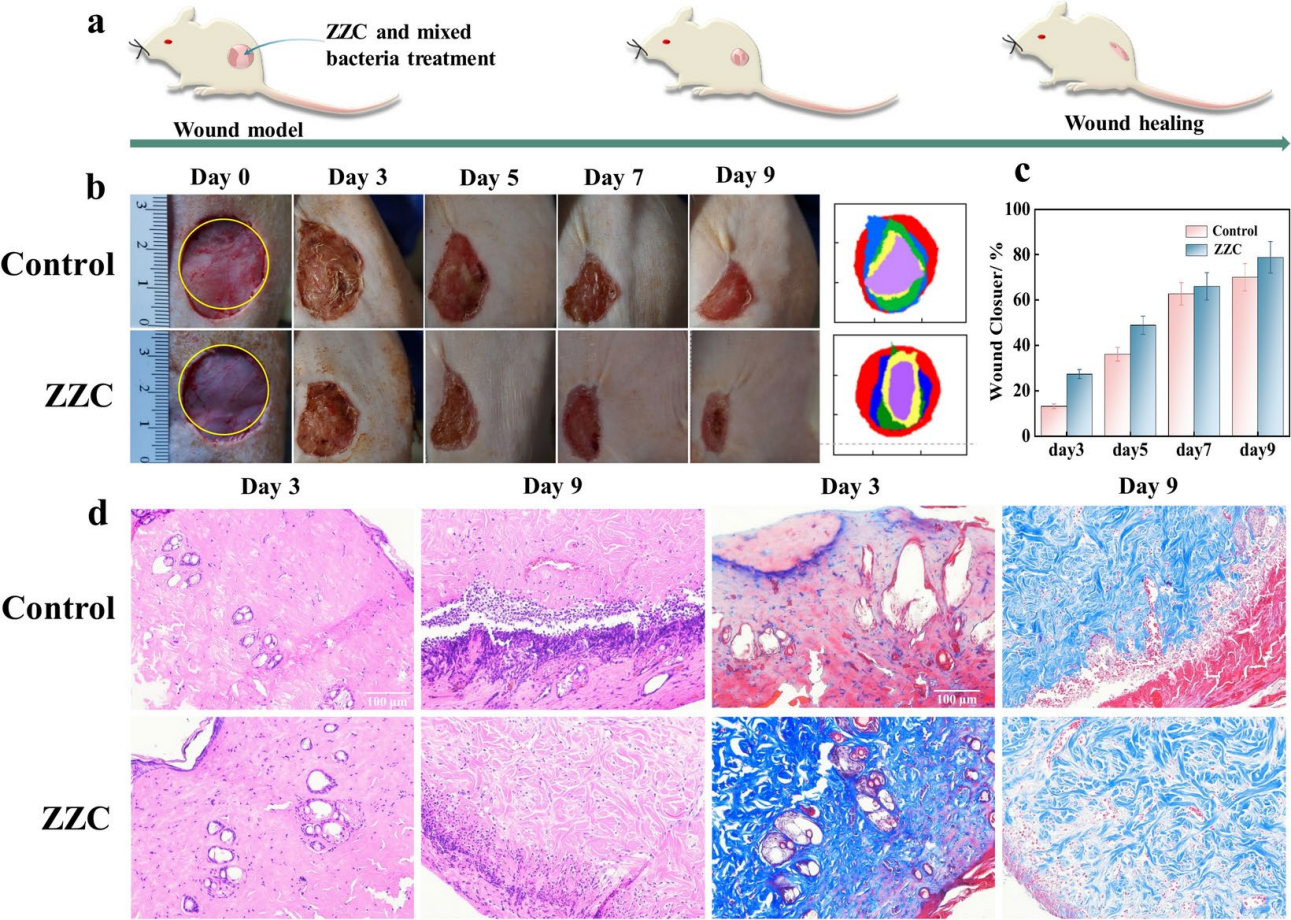
Schematic of ZZC-mediated treatment to inhibit bacteria and promote wound healing (**a**). Photographs of skin wounds infected with three bacterial mixtures on different days of treatment and superimposed wound healing diagram (**b**). Changes in skin wound area in different groups of mice (**c**). Dynamics of tissue recovery as shown by H&E staining and Masson staining histological images of ZZC on days 3 and 9 ninety days in the mixed bacterial wound infection mouse model. Scale bar is 100 μm (**d**).

Due to the good biocompatibility and bacteriostatic activity of ZZC, we inoculated the back wounds of SD rats with a bacterial infection model (Fig. 11a) and then investigated the antibacterial and wound healing properties of ZZC in vivo. Figure 11b shows the progression of wound healing and changes in crusting as well as a superimposed plot of the wound healing area of wounds infected with *E. coli*, *S. aureus* and *T-Salmonella* during 9 days of ZZC treatment. Figure 11c shows the relative size of the healed area of the wound at different times after treatment. PBS was unable to stop the mixed bacterial infections, resulting in large bacterial debris in the infected wounds. In contrast, the healing effect of ZZC was greater than that of PBS for the same number of days, with ZZC achieving a healing rate of 78.8% on day 9. This suggests that it has a better healing effect on mixed bacterial infected wounds. Infected tissues were collected from dorsal wounds and histological morphological changes were observed using hematoxylin–eosin (H&E) and Masson staining to assess the healing effect of ZZC. As shown in Fig. 11d, the ZZC group showed a significant reduction in inflammatory cells and the formation of more complete epidermal and pore structures in the wound tissue on day 9 compared with the control group, indicating that the skin was largely repaired. The Masson staining results showed that ZZC had excellent antimicrobial properties and promoted wound healing, and more collagen fibres were deposited in the ZZC group compared with the control group.

### Mechanism of bacterial inhibition by ZZC nanocomposites

**Fig. 12:**
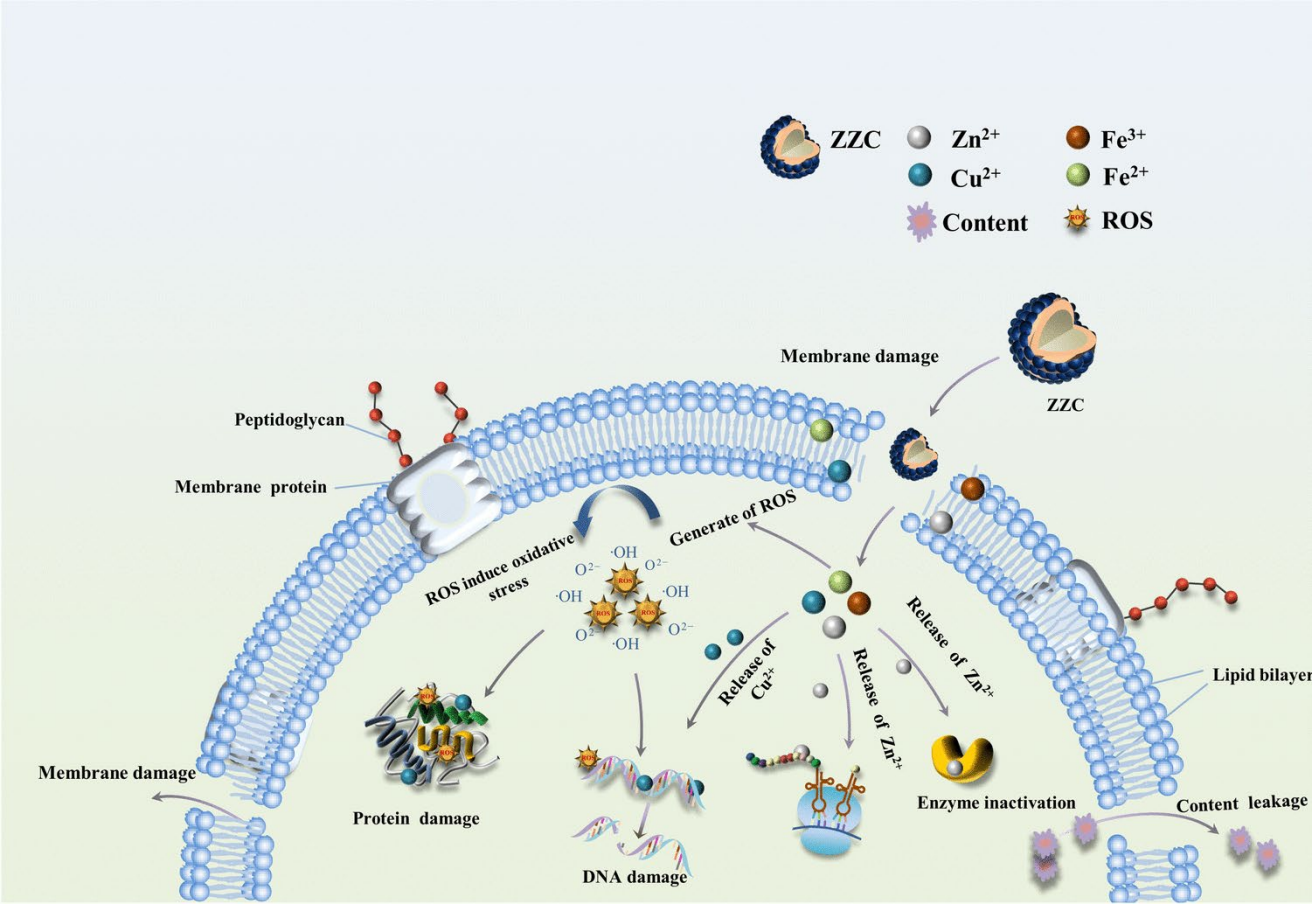
Schematic depicting the bacteriostatic mechanism.

In this study, we investigated the interactions between nano-ZZC composites and Gram-negative and Gram-positive bacteria using Zeta potential analysis, PI staining, and analysis of cytoplasmic leakage and proposed a possible mechanism of bacterial inhibition (Fig. 12). *E. coli* cell walls are approximately 10–15 nm and consist of phospholipids, lipopolysaccharides and proteins that form the outer membrane^47,48^, whereas the cell wall of *S. aureus* consists of a thick peptidoglycan (PGN) layer (20–80 nm), which is thicker and more compact, and interspersed with a large amount of phosphoglycolic acid^49,50^. The layer is surrounded by anionic glycopolymers, resulting in the cell walls having a negative potential difference due to the presence of –OH, –NH_2_, –COOH, –CONH_2_ and other groups. Cu^2+^, Zn^2+^and Fe^3+^ ions released from the ZZC nanocomposites during the interaction with the bacteria can be electrostatically adsorbed onto the cell membranes of Gram-negative bacteria, altering the secondary structures of proteins, and thus increasing the permeability of the membrane and ultimately resulting in the death of the microorganism. This is consistent with the results of the Zeta potential measurements. The ZZC nanocomposites material can cause bacterial death by disrupting the bacterial cell membrane ion channels, resulting in a massive leakage of K^+^, Ca^2+^and Mg^2+^ ions.

Essential metal ions, such as Cu, Zn and Fe, are involved in essential cellular functions, such as the synthesis of key enzymes and electron transport processes, as well as in the structure of cell membranes and DNA^51,52^. However, they can also have lethal effects on bacterial cells. For example, high concentrations of metal ion can negatively affect a variety of bacterial activities, such as glycolysis, transmembrane proton translocation and acid resistance, thereby prolonging the lag phase in bacterial growth^53^ and triggering metal mismatches in various metal-binding proteins, leading to protein dysfunction, enzyme inactivation, or protein denaturation, all of which disrupt the equilibrium of the bacterial cell. Zn^2+^ and Cu^2+^ ions released from ZZC can bind to DNA, disrupting its helical structure and leading to the death of the bacterial cell^54^; this is consistent with the results of the PI staining. Another possible mechanism may be through the generation of reactive oxygen species (ROS) through the Fenton reaction^55^. Under physiological conditions, iron exists mainly in two oxidation states, namely, the oxidized Fe^3+^ (trivalent iron) and the reduced Fe^2+^ (ferrous iron) forms. Fe^2+^ is easily oxidized, releasing electrons that can combine with oxygen to produce ROS^56^ while combination with Zn^2+^ not only increases electron mobility but also causes fatal damage to bacterial pathogens. Accumulation of ROS induces oxidative stress in the cell, potentially damaging bacterial membranes, ribosomes, proteins, and DNA 0.

## Conclusions

This study details the preparation of a new nanomaterial, ZnFe_2_O_4_, through the solvothermal method. A layer of ZIF-8 with a dodecahedral structure was used as the nucleus which was then coated with ZnFe_2_O_4_ is coated, resulting in the formation of ZZC nanocomposites via "layer-coating". The morphology of the ZZC nanocomposites was assessed using TEM, revealing the presence of core–shell spherical nanostructures on the surface.

The study investigated the bacteriostatic effects of the composites and found that they were significantly superior to those of available agrochemicals. The MIC of the ZZC against *E. coli*, *S. aureus* and *T-Salmonella* were 50, 60 and 80 μg/mL, respectively; At a concentration of 200 μg/mL, the composites displayed excellent antibacterial activity against *E. coli*, *S. aureus* and *T-Salmonella*, with inhibition rates for the three bacteria reaching 99.99% within 80 min. In vivo biological experiments demonstrated that the ZZC nanoparticles possessed substantial antibacterial efficacy, wound healing ability and favorable biocompatibility.

Based on the results of Zeta potential measurements, PI staining and cytoplasmic and ion leakage experiments, it is hypothesized that the composite material inhibits microbial growth by releasing Zn^2+^, Cu^2+^, Fe^3+^, negatively charged lipoproteins, and lipopolysaccharides from the cell wall through electrostatic attraction. This causes increased permeability of the microbial cell membrane and a change in the surface structure of the cell wall. Additionally, another possible mechanism involves the production of ROS which can cause oxidative stress within the cell, causing damage to proteins and DNA. This also impacts bacterial membranes, lipids, proteins and DNA, leading to oxidative stress. In summary, ZZC demonstrated remarkable bacteriostatic properties, providing a solid foundation for the future development of materials for medical treatments and other areas.

## Supplementary Information

Supplementary Information.

## Data Availability

Data will be made available on request. The point of contact for requesting data: 545355954@qq.com (Shaobo Guo), ZXLLF75@126.com (Xinli Zhou).
