# Supplementary material for: Study of antibacterial activity of copper zinc nanocomposites and disruption of bacterial cytoplasmic membrane
Source: Sci Rep. 2025 Jul 23;15:26780. doi: 10.1038/s41598-025-93691-1 (PMC12287259; doi:10.1038/s41598-025-93691-1)
Supplement: Supplementary file 1 — Supplementary Information. [file 41598_2025_93691_MOESM1_ESM.docx]

**Supplementary material**

**Study of antibacterial activity of copper zinc nanocomposites and disruption of bacterial cytoplasmic membrane**

*Zhongshang Guo^a^,* *Huihui Chen^b^, Ruiling Hu^b^, Jiawei Wang^b^,* *Miao Wu^b^, Yinghua Wu^b^, Tinghui Qiang^a^, Huan Mou^a^, Xingguo Du^a^, Fei Gao^a^, Shaobo Guo^b*^, Xinli Zhou^a*^*

^a^ Department of Osteoarticular Surgery Department, Hanzhong Central Hospital, Hanzhong 723000, Shaanxi, China.

^b^ Shaanxi Key Laboratory of Catalysis, School of Chemical & Environment Science, Shaanxi University of Technology, Hanzhong 723000, Shaanxi, China.

***Corresponding authors: 545355954@qq.com (Shaobo Guo), ZXLLF75@126.com (Xinli Zhou)**

**Figure 2**

**Page 2**

**Figure S1.** TEM image of ZZZC.

**Figure S2** Graph of MIC test results of nano-ZZ against *E. coli* (a), *S. aureus* (b), *T-Salmonella* (c)

**Figure S3** Graph of MIC test results of nano-ZZC against *E. coli* (a), *S. aureus* (b), *T-Salmonella* (c)

**Table S1** MIC values of nano-ZZC on *E. coli*, *S. aureus*,
*T-Salmonella*

| Bacteria | Material / Material concentration gradients c(μg/mL) / BD (1×10^8^ CFU/mL) | | | | | | | |  |  |
| --- | --- | --- | --- | --- | --- | --- | --- | --- | --- | --- |
|  | ZZC | | | | | | | |  |  |
|  | 0 | 10 | 20 | 30 | 40 | 50 | 60 | 70 | 80 | 90 |
| ***E. coli*** | 5.73 | 4.97 | 3.84 | 2.65 | 1.98 | **0.25** | 0.20 | 0.19 | 0.17 | 0.16 |
| ***S. aureus*** | 6.24 | 5.87 | 4.26 | 3.97 | 3.02 | 2.66 | **0.21** | 0.19 | 0.18 | 0.15 |
| ***T-Salmonella*** | 4.59 | 4.03 | 3.63 | 3.22 | 2.87 | 2.45 | 2.06 | 1.64 | **0.27** | 0.18 |

**Figure.S4**. Results of filter paper diffusion of different concentrations of zineb, Bordeaux solution, and ZZ and nano-ZZC composites against *S. aureus* (a), *E. coli* (b) and *T-Salmonella* (c). Curves showing the diameters of the bacteriostatic circles of different materials with different concentrations against *S. aureus*(d), *E. coli*(e) and *T-Salmonella*(f).

**Figure. S5**. Colony-counting results for *E. coli* (a), *S. aureus* (b) and *T-Salmonella* (c) with nano-ZZC composites at a concentration of 200 µg/mL.

**Figure. S6**. The surface potential of the ZZC composites

**Figure. S7**. Cytoplasmic leakage of *S. aureus* (a), *E. coli* (b) and *T-Salmonella* (c) after treatment with the ZZ nanocomposites.

**Figure. S8**. The IC_70_ value of the ZZC composites.
